# Tumor Mutational Burden Determined by Panel Sequencing Predicts Survival After Immunotherapy in Patients With Advanced Gastric Cancer

**DOI:** 10.3389/fonc.2020.00314

**Published:** 2020-03-13

**Authors:** Jinchul Kim, Binnari Kim, So Young Kang, You Jeong Heo, Se Hoon Park, Seung Tae Kim, Won Ki Kang, Jeeyun Lee, Kyoung-Mee Kim

**Affiliations:** ^1^Department of Medicine, Division of Hematology-Oncology, Samsung Medical Center, Sungkyunkwan University School of Medicine, Seoul, South Korea; ^2^Department of Pathology and Translational Genomics, Samsung Medical Center, Sungkyunkwan University School of Medicine, Seoul, South Korea; ^3^The Samsung Advanced Institute for Health Sciences & Technology (SAIHST), Samsung Medical Center, Sungkyunkwan University School of Medicine, Seoul, South Korea

**Keywords:** tumor mutational burden, panel sequencing, cut-off points, immune checkpoint blockade, gastric cancer

## Abstract

**Objective:** Panel-based sequencing is widely used to measure tumor mutational burden (TMB) in clinical trials and is ready to enter routine diagnostics. However, cut-off points to distinguish “TMB-high” from “TMB-low” tumors are not consistent and the clinical implications of TMB in predicting responses to immune checkpoint blockade (ICB) in gastric cancer are not clearly defined. We aimed to assess whether TMB is associated with the response to immunotherapy and to examine its relation with other biomarkers of immunotherapy response in advanced gastric cancer.

**Design:** In total, 63 patients with advanced gastric cancer treated with ICB were included in the study. Panel-based TMB in gastric tumor samples, treatment responses to ICB, clinicopathological data, and time to progression were retrospectively analyzed. Microsatellite instability (MSI) status, Epstein–Barr virus (EBV) positivity, and programmed death-ligand 1 (PD-L1) combined positive score (CPS) were also analyzed.

**Results:** TMB ranged from 0 to 446 mutations/megabase (mt/mb) and was significantly associated with MSI (*P* < 0.001), PD-L1 CPS (*P* = 0.022), response to ICB (*P* = 0.04), chemotherapy (*P* = 0.02) and older patient age (≥65 years; *P* = 0.0014). The cut-off point of 14.31 mt/mb determined by log-rank statistics for progression-free survival divided the tumors into eight (12.7%) TMB-high and 55 (87.3%) TMB-low tumors. The median TMB of the chemo-refractory group was significantly higher (8.43 mt/mb) compared to that of chemo-naïve group (3.42 mt/mb) (*P* = 0.02). Patients with TMB-high tumors showed prolonged progression-free survival in univariate [HR, 0.32; 95% confidence interval (CI), 0.12–0.90] and multivariate (HR, 0.21; 95% CI, 0.07–0.69) analyses. In area under the receiver operating curve (AUC) analysis of TMB, PD-L1, EBV, MSI, and their combination, the AUC value was the highest for EBV (0.97), followed by MSI (0.96), PD-L1 (0.81), the combination (0.78), and TMB (0.56).

**Conclusion:** In addition to EBV, MSI, and PD-L1 CPS, TMB could be used as a predictive biomarker in patients with advanced gastric cancer treated with ICB and may aid clinical decision making.

## Introduction

Immunotherapy has emerged as an innovative treatment for several types of cancer (1–6). Cancer cells can escape immune surveillance by upregulating immune-checkpoint proteins, such as programmed death ligand 1 (PD-L1) (7). This escape can be counteracted by using immune-checkpoint blockade (ICB), such as pembrolizumab and nivolumab, which interrupt the interaction between programmed cell death-1 (PD-1) and PD-L1. ICB have been approved for various types of cancer, including non-small-cell lung cancer, melanoma, and renal cell carcinoma (1–6), and recently also for previously treated advanced gastric cancer with PD-L1 expression (combined positive score (CPS) ≥1) (8, 9). However, clinical trials using ICB have reported a wide range of response rates (10–26%) in patients with advanced gastric cancer without a clear selective biomarker or PD-L1 positivity (8–10).

Gastric cancer is a complex and heterogeneous disease. Gastric tumors are classified into four molecular subgroups: tumors positive for Epstein–Barr virus (EBV), microsatellite unstable (MSI) tumors, genomically stable tumors (GS), and tumors with chromosomal instability (CIN) (11). Although ICB have durable antitumor activity and improve survival in a subset of patients with PD-L1-expressing, MSI, and EBV-positive tumors (11, 12), there is a need for additional predictive biomarkers to improve patient selection and avoid toxicity in potential non-responders (13).

Tumor mutational burden (TMB) has been emerged as a new biomarker for PD-L1 antibody treatment in diverse tumor types (14–16). However, its predictive effect in advanced gastric cancer had not yet been demonstrated (17). Recently, Wang et al. (18) have identified TMB as a biomarker for survival benefit in chemo-refractory gastric cancer treated with toripalimab: High TMB group showed significant superior overall survival (OS) than the TMB-low group (14.6 vs. 4.0 months, HR = 0.48, *P* = 0.038). TMB has been traditionally determined by whole exome sequencing; however, high cost and lengthy turnaround time limit its widespread use in clinic (12, 13, 19, 20). Current precision oncology platforms generally use next-generation sequencing of targeted gene panels (14). However, most studies determining TMB by panel-based sequencing were performed in patients with non-small-cell lung cancer or melanoma (13, 16, 20). Although several studies evaluated TMB in gastric cancer, most studies proposed cut-off points for TMB and their relevance to survival (21–23), and only two studies included patients treated with ICB, in which TMB was measured by whole exome sequencing (18) and custom-designed large-sized panels (14). Moreover, despite efforts to standardize the TMB from multiple different genomic profiling panels (24), the cut-off value for TMB is not consistent and the associations with other biomarkers have not been fully defined in gastric cancer. For the use in daily clinical practice, a smaller and standardized panel should be developed and needs to be validated in clinical trials (17).

The present study aimed to assess the clinical implications of TMB using a small and verified panel and established biomarkers (PD-L1 CPS, EBV, and MSI) to better characterize and select advanced gastric cancer patients who may benefit from immunotherapy.

## Materials and Methods

### Patients and Data Collection

Data of 81 patients with advanced gastric cancer and metastasis treated with an ICB (pembrolizumab or nivolumab) at Samsung Medical Center between December 2016 and January 2019 were retrospectively collected. One patient was excluded because of follow-up loss and 17 patients were excluded because TMB could not be assessed because of a lack of tissue. Finally, 63 patients with metastatic gastric cancer were included in the study.

Median age was 54 years (29–82), and 37 (58.7%) patients were male. Clinicopathological data were retrospectively extracted from electronic medical records. Patients were evaluated for treatment response by chest or abdominal/pelvic computed tomography. The response rate was assessed using RECIST 1.1 criteria (25). The biopsy specimens were all from archival formalin-fixed paraffin-embedded tissues, of which 58 (92.1%) were obtained prior to ICB therapy. 58 (92.1%) were obtained from the primary tumors, and 46 (73%) tissues were obtained before chemotherapy (Table S1). This study was performed in accordance with institutional review board guidelines for data analysis and investigational treatments, and patient consent was obtained.

### Sample Preparation and DNA Extraction

Tumor-rich areas for dissection were circled by pathologists on haematoxylin and eosin-stained 5-μm-thick slides from formalin-fixed, paraffin-embedded (FFPE) tissue. The tumor cell percentages with >20% on H&E-stained slides were selected. The sections were macro-dissected along the circle and deparaffinized using xylene and 100% ethanol. Genomic DNA was extracted using the ReliaPrep^TM^ FFPE Total DNA Miniprep System (Promega, Madison, WI). Nucleic acids were quantified using the Qubit dsDNA HS Assay Kit and the amount of amplifiable DNA by qPCR.

### Sequencing

All 63 cases were evaluated using the targeted high-throughput sequencing Oncomine Comprehensive Assay v3 (Thermo Fisher Scientific), which enables simultaneous detection of hundreds of variants across 161 genes relevant to solid tumors (Table S2). Libraries were prepared and were sequenced on an Ion S5 XL Sequencer using the Ion 540 Kit-Chef. Raw sequence data were analyzed using Ion Torrent software (Ion Reporter 5.6) and Oncomine Knowledgebase Reporter (all from Thermo Fisher Scientific). The reads were aligned to Genome Reference Consortium Human Build 37 (hg19) within Torrent Suite, and a minimum average read depth of 1,000 was required for analysis. Tier I or II genetic alterations were identified according to standards and guidelines for the interpretation and reporting of sequence variants in cancer (26). Briefly, the criterion of variant allele frequency for single nucleotide variants (SNVs)/insertions and deletions (indels) was ≥5%. Average copy number ≥4 was interpreted as a gain (amplification) and <1 as a loss (deletion). Most tumor samples were within the standards of sequencing results, such as mapped reads >5,000,000, on-target rate >90%, mean depth >1,200, and uniformity >90%. The final analysis of each case was reviewed and reported by a pathologist (K-MK) in a College of American Pathologists (CAP)-certified laboratory. All the sequencing data have been deposited into European Nucleotide Archive (ENA) (Webin-53995).

### TMB Assay

The Oncomine Tumor Mutation Load Assay was used to assess TMB (somatic mutations per Mb) by interrogating 409 key cancer genes (Table S3). This assay counts SNVs including both non-synonymous and synonymous mutations from coding and non-coding regions. The analysis pipeline calls variants with optimized parameters on the tumor sample only, with no requirement for matched normal samples, and applies filters to remove germline variants (27). Libraries were prepared using Oncomine Tumor Mutation Load Assay library preparation kits (Chef-Ready) and barcoded adapters were ligated. Eight libraries were combined and loaded onto a single Ion 540 Chip. Reads were aligned to hg19 using Torrent Suite 5.6, and BAM files were transferred to Ion Reporter 5.6 for variant calling and secondary analysis, including TMB calculation.

### EBV Detection

Three-micrometer-thick sections were cut from FFPE tissue blocks and mounted on SuperFrost Plus slides (Thermo Fisher Scientific). The BOND-MAX system was used for *in-situ* hybridization (ISH) with an EBV-encoded RNA probe (Leica, Newcastle, UK). Only nuclei of tumor cells with strong brown signals were considered positive.

### MSI PCR and Immunohistochemistry for Mismatch Repair Proteins

MSI status was determined by multiplex PCR to amplify five quasimonomorphic mononucleotide repeat markers (BAT-25, BAT-26, NR-21, NR-24, and NR-27). Genomic DNA was isolated from FFPE tissue blocks using a QIAamp DNA Mini Kit (Qiagen, Hilden, Germany). Sense primers were fluorescently end-labeled with FAM, HEX, or NED. Amplicons were analyzed on an ABI Prism 3130 Genetic Analyzer (Applied Biosystems, Foster City, CA). Allelic sizes were estimated with GeneMapper 4.1 (Applied Biosystems), and tumors with allelic size variation in <2 and ≥2 microsatellites were classified as microsatellite stable (MSS) and instable, respectively.

For mismatch repair protein immunohistochemistry, antibodies against MLH1 (clone ES05; 1:100 dilution; Novocastra) and MSH2 (clone G219-1129; 1:500 dilution; Cell Marque) were used, as previously reported (23).

### PD-L1 Immunohistochemistry

FFPE tissue blocks were cut into 4-μm sections and were stained on an Autostainer Link 48 system (Agilent Technologies) using a Dako PD-L1 IHC 22C3 pharmDx kit (Agilent Technologies) and visualized using EnVision FLEX, as previously described (12). The CPS of PD-L1 expression was calculated as the number of PD-L1-stained tumor and immune cells divided by total number of viable tumor cells, multiplied by 100. Cases with CPS ≥1 were considered positive.

### Data Analysis

Statistical tests included two-sample tests of proportions, Fisher's exact test, two-sample tests for continuous variables that did not follow a normal distribution, and Wilcoxon rank-sum test.

Kruskal-Wallis test with Dunn correction was used for multiple comparisons for three or more groups. Spearman's correlation was used to examine the association between PD-L1 CPS and TMB. The optimal cut-off point for dividing patients into TMB-high and TMB-low groups was determined using log-rank statistics in the R package “maxstat.” The Kaplan–Meier method and log-rank test in the “survival” R package were used to compare progression-free survival (PFS) between TMB-high and TMB-low groups. Uni- and multivariate Cox regression analyses were utilized to assess hazard ratios and confidence intervals according to TMB and several other clinicopathologic factors. Two-sided *P* ≤ 0.05 were considered significant. All statistical analyses were performed using R version 3.5.3.

To evaluate the biomarker potential of TMB and PD-L1, EBV, and MSI to predict responses to ICB, areas under the receiver operating characteristic (ROC) curve (AUCs) were determined using the ROCR package (URL http://rocr.bioinf.mpi-sb.mpg.de). Immunotherapy prediction models were constructed based on PFS using Cox regression and based on RECIST response rate using linear regression. Cut-off points for the Cox model were calculated by comparing PFS with maximally selected rank statistics using maxstat. The linear regression model was calculated using the accuracy function in the AUC package (https://CRAN.R-project.org/package=AUC). To evaluate these models, AUCs were calculated using the survivalROC and RORC packages.

## Results

### Patient Characteristics

Clinical data of 63 patients with advanced gastric cancer treated with ICB and quantifiable TMB were evaluated. The patients were treated with nivolumab (*n* = 29) or pembrolizumab (*n* = 34). Thirteen patients showed a complete response (CR) or partial response (PR), and 50 patients were non-responders with stable disease (SD) or progressive disease (PD). TMB values ranged from 0 to 446 mt/mb. The median TMB was 4.23. The optimal cut-off point for dividing patients into TMB-high and -low groups based on log-rank statistics for PFS was 14.31 mt/mb. Based on this cut-off point, eight patients (12.7%) had TMB-high tumors. Twenty-six (41.3%) patients showed PD-L1 positivity. Most patients (80.9%) had tubular adenocarcinoma, but two patients had adenocarcinoma with neuroendocrine differentiation. Forty-four patients (69.9%) had poorly differentiated tumors, and 39 (61.9%) patients underwent gastrectomy. Twenty-three (36.5%) and 24 (38.1%) patients received ICB as a third- or later-line and as a second-line treatment, respectively. Fifty (79.4%) patients had peritoneal carcinomatosis when immunotherapy was started (Table 1).

**Table 1:** Patient characteristics.

	**CR/PR (*N* = 13)**	**SD/PD (*N* = 50)**	**Total (*N* = 63)**
**Sex**			
-F	3 (23.1%)	23 (46.0%)	26 (41.3%)
-M	10 (76.9%)	27 (54.0%)	37 (58.7%)
Age median value (range)	66 (32–82)	53 (29–72)	54 (29–82)
**EBV**			
-Negative	10 (76.9%)	46 (92.0%)	56 (88.9%)
-Positive	2 (15.4%)	2 (4.0%)	4 (6.3%)
-Unknown	1 (7.7%)	2 (4.0%)	3 (4.8%)
**TMB median value (range)**	7.58 (0–446)	3.42 (0–169)	4.23 (0–446)
-High	4 (30.8%)	4 (8.0%)	8 (12.7%)
-Low	9 (69.2%)	46 (92.0%)	55 (87.3%)
**MSI status**			
-MSI	5 (38.5%)	1 (2.0%)	6 (9.5%)
-MSS	8 (61.5%)	49 (98.0%)	57 (90.5%)
**PD-L1**			
-Negative	5 (38.5%)	31 (62.0%)	36 (57.1%)
-Positive	8 (61.5%)	18 (36.0%)	26 (41.3%)
-Unknown	0 (0.0%)	1 (2.0%)	1 (1.6%)
**ECOG PS**			
-0–1	11 (84.6%)	37 (74.0%)	48 (76.2%)
-2–3	2 (15.4%)	13 (26.0%)	15 (23.8%)
**Pathologic subtype**			
-TADC	10 (76.9%)	41 (82.0%)	51 (80.9%)
-SRC	1 (7.7%)	9 (18.0%)	10 (15.9%)
-NED	2 (15.4%)	0 (0.0%)	2 (3.2%)
**Differentiation**			
-WD	2 (15.4%)	3 (6.0%)	5 (7.9%)
-MD	3 (23.1%)	11 (22.0%)	14 (22.2%)
-PD	8 (61.5%)	36 (72.0%)	44 (69.9%)
**Previous gastrectomy**			
-No	5 (38.5%)	19 (38.0%)	24 (38.1%)
-Yes	8 (61.5%)	31 (62.0%)	39 (61.9%)
**Previous line of treatment**			
-0	1 (7.7%)	1 (2.0%)	2 (3.2%)
-1	5 (38.5%)	9 (18.0%)	14 (22.2%)
-2	4 (30.8%)	20 (40.0%)	24 (38.1%)
-≥3	3 (23.1%)	20 (40.0%)	23 (36.5%)
**Tumor site**			
-Antrum	6 (46.1%)	11 (22.0%)	17 (27.0%)
-Body/angle	4 (30.8%)	34 (68.0%)	38 (60.3%)
-Cardia/GEJ	3 (23.1%)	3 (6.0%)	6 (9.5%)
-Fundus	0 (0.0%)	1 (2.0%)	1 (1.6%)
-Whole stomach	0 (0.0%)	1 (2.0%)	1 (1.6%)
**HER2 positivity**			
-Negative	10 (76.9%)	39 (78.0%)	49 (77.8%)
-Positive	1 (7.7%)	3 (6.0%)	4 (6.3%)
-Unknown	2 (15.4%)	8 (16.0%)	10 (15.9%)
**Peritoneal carcinomatosis**			
-No	7 (53.8%)	6 (12.0%)	13 (20.6%)
- Yes	6 (46.2%)	44 (88.0%)	50 (79.4%)
**Checkpoint inhibitor**			
-Nivolumab	3 (23.1%)	26 (52.0%)	29 (46.0%)
-Pembrolizumab	10 (76.9%)	24 (48.0%)	34 (54.0%)

*CR, complete response; PR, partial response; SD, stable disease; PD, progressive disease; EBV, Epstein-Barr virus; TMB, tumor mutational burden; MSI, microsatellite instability; MSS, microsatellite-stable; PD-L1, programmed death-ligand 1; ECOG PS, Eastern Cooperative Oncology Group performance status; TADC, tubular adenocarcinoma; SRC, signet ring cell carcinoma; NED, adenocarcinoma with neuroendocrine differentiation; WD, well differentiated; MD, moderately differentiated; PD, poorly differentiated; GEJ, gastro-esophageal junction*.

### Correlations Between TMB and Other Biomarkers

**Figure 1:**
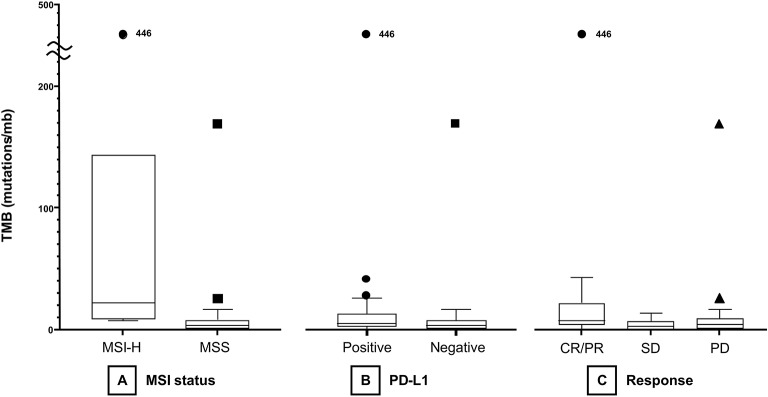
Boxplots of TMB according to MSI status **(A)**, PD-L1 positivity **(B)**, and treatment response **(C)**. TMB, tumor mutational burden; MSI, microsatellite instability; MSS, microsatellite-stable; PD-L1, programmed death-ligand 1; CR, complete response; PR, partial response; SD, stable disease; PD, progressive disease.

The median TMB of the MSI group (21.93 mt/mb) was significantly higher than that of the MSS group (3.42 mt/mb, *P* < 0.001, Figure 1A). Median TMB was higher in the PD-L1-positive (5.24 mt/mb) than in the PD-L1 negative (3.42 mt/mb) group, with marginal statistical significance (*P* = 0.05, Figure 1B). However, TMB showed a significant association with PD-L1 CPS as a continuous variable (Spearman's correlation coefficient = 0.30, *P* = 0.022, Figure S1). TMB was significantly different among responders (CR/PR), SD, and PD groups (median 7.58 vs. 2.94 vs. 4.22 mt/mb) (*P* = 0.04) (Figure 1C). In Dunn *post-hoc* analysis, TMB was significantly higher in patients with CR/PR than in those with SD (*P* = 0.04), while TMB in CR/PR was not significantly different with TMB in PD group (*P* = 0.14).

The median TMB of the chemo-refractory group was significantly higher (8.43 mt/mb) compared to that of chemo-naïve group (3.42 mt/mb) (*P* = 0.02) (Table S4).

**Figure 2:**
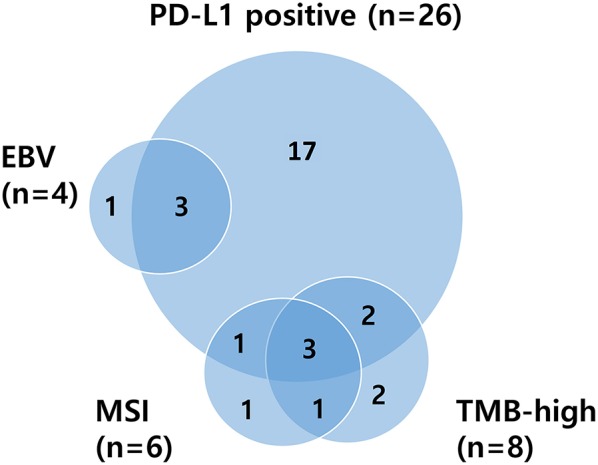
Venn diagram illustrating relationships between PD-L1 positivity, MSI status, EBV positivity, and TMB. PD-L1, programmed death-ligand 1; MSI, microsatellite instability; EBV, Epstein–Barr virus; TMB, tumor mutational burden.

Patients of ≥65 years showed significantly higher TMB values than younger patients (median 9.39 vs. 3.38 mt/mb; *P* = 0.0014). Overall, age ≥65 (*P* = 0.0014), MSI (*P* < 0.001), and responders (CR/PR) (*P* = 0.04) were significantly associated with TMB-high (Table S4). Relations between PD-L1 positivity, MSI status, EBV-positivity, and TMB are depicted in a Venn diagram in Figure 2. There was substantial overlap between MSI and TMB-high tumors, whereas only some PD-L1-positive tumors were also MSI or TMB-high. The median TMB of EBV-positive patients was higher than that of EBV-negative patients (5.06 vs. 3.81 mt/mb), but all EBV-positive patients (*n* = 4) were TMB-low, and three of them showed PD-L1 positivity.

### Correlation of Response to PD-1 Inhibitors With Molecular Subtype

To further evaluate biomarkers of ICB, response rates in modified molecular subtypes of gastric cancer were compared (28). Tumors were categorized into MSI (*n* = 6), EBV (*n* = 4), CIN (with *TP53* mutation or copy number alteration; *n* = 23), TP53^+^GS^−^ (SNVs in genes other than *TP53*; *n* = 6), and GS (no genetic alteration; *n* = 24). Patients with MSI tumors (median, 74.5 years) were the oldest. All patients with EBV-positive and MSI gastric cancer were male. PD-1 positivity was the highest in the EBV group (75%) and the lowest in the GS group (20.8%). Median TMB was the highest in the MSI group (21.92) and the lowest in the GS group (2.12). Clinicopathological factors according to molecular subtypes are presented in Table S5.

### Non-responders With Favorable Biomarkers (EBV-Positive/MSI/TMB-High)

Of the six patients with MSI gastric cancer, five achieved PR with a median PFS of 12 months and one was refractory to nivolumab with a PFS of 0.17 months. The non-responder was a 72-years-old male with EBV-negative, PD-L1-positive, and TMB-low (10.11 mt/mb) cancer. Of the four patients with EBV-positive gastric cancer, two achieved PR with a PFS of 7.2 and 12.7 months and two were refractory to pembrolizumab and nivolumab with a PFS of 1.4 and 0.3 months. These non-responders were male and had TMB-low (4.23 and 9.32 mt/mb) and MSS tumors; one case was PD-L1 negative. Of the eight patients with high TMB, four were responders with median a PFS of 12.05 months and four were non-responders with a median PFS of 2.4 months. These non-responders were male, had MSS tumors, were EBV-negative, and had a median TMB of 21.65 mt/mb, slightly lower than that of responders with TMB-high tumors (35.27 mt/mb). In non-responders with TMB-high, PD-L1 positivity was found in 50% of cases.

### Responders Without Favorable Biomarkers (EBV-Negative/MSS/TMB-Low)

There were five responders without any favorable biomarkers including EBV-positivity, MSI, and TMB-high. Patient no. 18 was a 49-years-old female with TMB of 3.38 mt/mb and PD-L1 positivity. The tumor was CIN subtype with co-amplification of *ERBB* and *MYC*. Treatment duration was 14.2 months and PFS was 14.9 months. Patient no. 36 was a 44-years-old female with a TMB of 9.27 mt/mb and PD-L1 negativity. The tumor had an *ARID1A* nonsense mutation and was classified as TP53^+^GS^−^ subtype. Treatment duration was 0.56 months, and PFS was 3 months. Patient no. 63 was a 32-years-old female with no TMB (0 mt/mb) and PD-L1 negativity. The tumor was GS subtype and the treatment duration and PFS were 9.63 months. Patient no. 77 was a 64-years-old male with a TMB of 6.75 mt/mb and PD-L1 positivity. The tumor was TP53^+^GS^−^ subtype, with *PIK3R1* mutation. Treatment duration was 3.6 months and PFS was 3.13 months. Patient no. 81 was a 55-years-old male with a TMB of 3.38 mt/mb and PD-L1 negativity. The tumor was CIN subtype with *TP53* mutation. Treatment duration was 5.36 months and PFS was 5.6 months. The two male patients had adenocarcinoma with neuroendocrine differentiation and patient no. 81 showed residual carcinoma in several metastatic lymph nodes in a gastrectomy specimen after therapy. Although several patients experienced PR to ICB without favorable biomarkers, most of them eventually progressed in <6 months.

### TMB as a Predictive Marker for Objective Response and PFS

**Figure 3:**
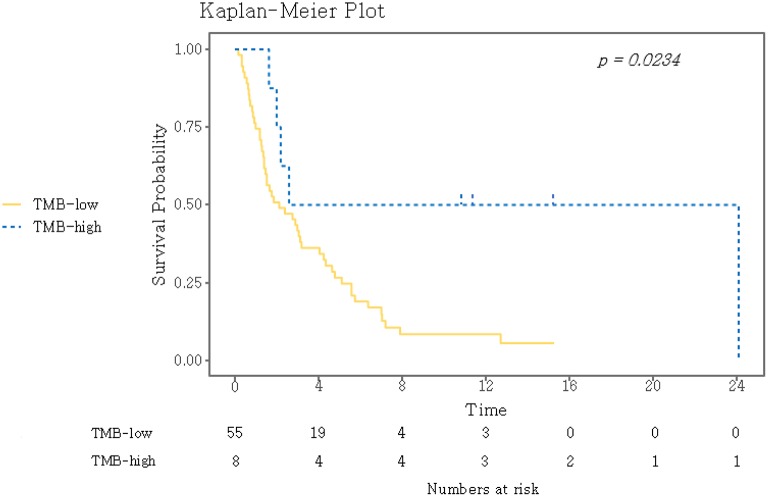
Kaplan–Meier curves for PFS for the dichotomized TMB groups. A log-rank test was used to compare the two groups. TMB, tumor mutational burden.

**Figure 4:**
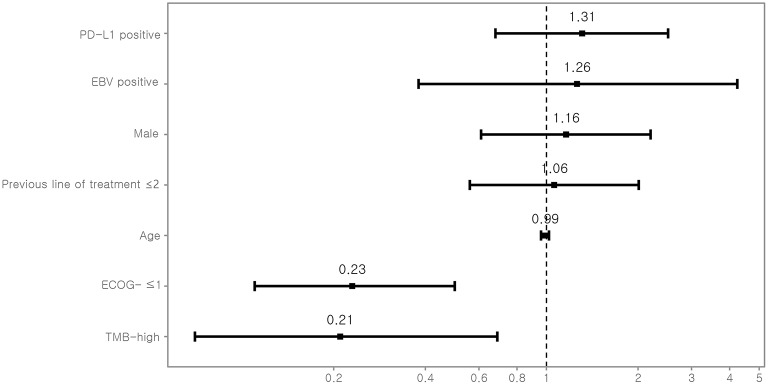
Multivariate Cox-regression analysis for PFS. For each variable, the estimated hazard ratio with 95% confidence interval is shown. PD-L1, programmed death-ligand 1; EBV, Epstein-Barr virus; ECOG, Eastern Cooperative Oncology Group performance status; TMB, tumor mutational burden.

TMB was assessed as a potential predictor for objective response and PFS. Eight (12.7%) patients were classified as TMB-high and 55 (87.3%) as TMB-low. Kaplan–Meyer survival curve analysis corroborated that TMB-high was significantly associated with longer PFS (*P* = 0.02, Figure 3). Univariate Cox analysis showed that the independent variables TMB group, MSI status, treatment response, and Eastern Cooperative Oncology Group (ECOG) performance status were significantly associated with PFS (Table S6). However, PD-L1 positivity was not significantly associated with PFS (*P* = 0.530). Multivariate analysis using stepwise Cox regression modeling including age, sex, EBV, TMB (categorized), PD-L1 positivity, ECOG performance status (≤1 vs. >1), and previous line of treatment (≤2 vs. >2) was performed. These parameters were included because they were considered to potentially significantly influence the response to immunotherapy. ECOG ≤1 (HR = 0.23; 95% CI, 0.11–0.50, *P* < 0.001) and TMB-high (HR = 0.21; 95% CI, 0.07–0.69, *P* = 0.01) were independent predictors of significantly prolonged PFS (Figure 4). The usability of TMB as a predictive biomarker for immunotherapy responses was further evaluated by AUC analyses of each biomarker (TMB, PD-L1, EBV, and MSI) (Figure S2A) and their combination (Figure S2B) based on 6-months PFS. The AUC value was the highest for EBV (0.97), followed by MSI (0.96), PD-L1 (0.81), the combination (0.78), and TMB (0.56).

## Discussion

Here, we analyzed TMB determined based on panel sequencing in 63 patients with advanced gastric cancer treated with ICB, correlated the clinical outcomes with TMB, and assessed its usability as a biomarker to predict response to ICB.

In our study cohort, ECOG ≤1 and TMB-high were independent predictors of prolonged PFS after treatment with ICB. In a previous study on patients with metastatic gastric cancer treated with pembrolizumab, circulating tumor DNA mutation load was slightly, albeit not significantly correlated with objective response and predicted PFS (12). Like PD-L1 expression, the thresholds to define high TMB vary depending on the technique used and tumor type (13, 24). Factors that influence TMB include tumor type, pre-analytic variables, the method used (whole exome vs. panel sequencing), and bioinformatics (20, 24). A previous study estimated TMB from FFPE tissues of diverse cancer types with a commercially targeted gene panel and categorized TMB as low (1–5 mt/mb), intermediate (6–19 mt/mb), or high (≥20 mt/mb) (16). In a study on 126 patients with esophagogastric cancer using an upper 20 percentile cut-off for TMB (8.8 mt/mb) to distinguish TMB-high and -low groups, TMB could predict clinical response to ICB (14). Recent clinical study in advanced gastric cancer patients investigating the safety and efficacy of toriparimab, a newly approved humanized PD-1 antibody, showed that high TMB (12 mt/mb, upper 20 percentile cut-off) was associated with improved patient responses and survival benefit after immunotherapy (18). In our study, a TMB cut-off of 14.31 mt/mb dichotomized patients into TMB-high and -low groups; eight patients were TMB-high and 55 were TMB-low. The TMB-high subgroup was significantly associated with higher objective response rate and prolonged PFS after ICB therapy, and with MSI, PD-L1 CPS, response to ICB, and older patient age, as expected. When we set the cut-off at the upper 20 percentile (10.6 mt/mb), although patients with TMB-high still showed higher ORR (38.5 vs. 16%) and prolonged PFS (2.6 vs. 2.3 months) after ICB therapy, the differences were not significant. For TMB, recent systematic literature review of 81 prior publications concluded that standardized threshold for classifying TMB levels as low- and high- does not exist currently (29). In a large-scale study (*n* = 1,662) analyzing the association between TMB and clinical responses to checkpoint inhibitors in various cancer types (14), they used a top-20th percentile as a cutoff for TMB and suggested that that there may not be one universal definition of high TMB. To set a TMB cut-off point, flexible criteria are recommended depending on the cancer type and sequencing panel used. The panel sizes and TMB cut-off values in previous trials are summarized in Table S7. The present TMB study with commercially available, small, and verified NGS panel will inform or guide oncologists in therapeutic decision making and will improve interpretability of TMB data across many assays.

Potential biomarkers were further analyzed for their predictability by AUC analyses. Although EBV, MSI, and PD-L1 predicted PFS more accurately than did TMB, the latter also predicted PFS. Many studies have reported limitations of PD-L1 as a biomarker of immunotherapy response based on ORR (8–10), and responders generally have longer PFS. These discrepant findings might be explained by the different methods used to measure ORR and PFS. Additionally, when biomarker predictability for ORR was evaluated, the AUC values were 0.62, 0.62, 0.56, and 0.70 for TMB, PD-L1, EBV, and MSI, respectively. To identify whether ORR or PFS is a better indicator of patient prognosis after immunotherapy, clinical trials in a gastric cancer cohort are needed.

Clinicopathological factors associated with TMB were also evaluated. Older age, MSI, PD-L1 CPS, chemotherapy and objective responses were significantly associated with TMB. Although the number of cases is small, the results are consistent with previous studies (16, 21, 22). We also observed that the median TMB of EBV-positive gastric cancer cases was higher than that of EBV-negative group, although it did not reach statistical significance as previously described (12). Although the direct relationship between EBV and TMB is unclear at the present time, EBV induces hypermethylation of CpG islands in the promoter regions of tumor-suppressor genes and affects miRNA levels (30), therefore, EBV may increase the TMB level, but not as much as MSI.

To further evaluate response predictors, response to ICB was correlated with gastric cancer molecular subtypes (11). Gastric cancers were divided into five subtypes, including the TP53^+^GS^−^ subtype. The response rate was associated with the molecular subtype, and these observations are consistent with those in our previous study (12), except for a lower response rate to ICB in EBV-positive tumors. Given the association of *ARID1A* alteration with EBV and MSI, and responses to ICB in gastric cancer (11), we further investigated *ARID1A*. All three tumors with *ARID1A* mutations were TP53^+^GS^−^ subtype and one patient showed PR while the other two patients showed PD.

The median TMB was also related with the response rate and molecular tumor subtype: a high median TMB (21.92 mt/mb) and high ORR (83.3%) were found in the MSI subtype, whereas a very low median TMB (2.12 mt/mb) and low ORR (4.2%) were found in the GS subtype. The median TMB of TP53^+^GS^−^ tumors was slightly higher than that of EBV-positive tumors, but the response rate of TP53^+^GS^−^ tumors was lower than that of EBV-positive tumors.

Limitations of the present study include (1) the small study cohort and its retrospective design. The cutoff values determined by random selection of patients and performing multiple classifications would result in large variations, warranting further validation in a large cohort with generalized dataset. (2) We used RECIST criteria instead of iRECIST criteria. Actually, a few patients progressed rapidly on ICB and we cannot exclude the possibility of pseudoprogression. (3) Although the effectiveness of Oncomine Tumor Mutation Load Assay's germline filtering has been verified (27), we did not use non-tumorous control samples.

Despite its significance in the immuno-oncological field, this is the first study to show the prognostic value of TMB for survival. High TMB was associated with improved PFS even after adjustment for clinically important variables, such as sex, age, EBV, ECOG performance status, PD-L1 positivity. In conclusion, TMB has potential as a predictive biomarker in patients with advanced gastric cancer treated with ICB and may be useful for clinical decision making in addition to EBV, MSI, and PD-L1.

## Data Availability Statement

The datasets generated for this study are available on request to the corresponding author.

## Ethics Statement

The studies involving human participants were reviewed and approved by Institutional Review Board of Samsung Medical Center (Seoul, Korea). The patients/participants provided their written informed consent to participate in this study.

## Author Contributions

All authors listed have made a substantial, direct and intellectual contribution to the work, and approved it for publication.

### Conflict of Interest

The authors declare that the research was conducted in the absence of any commercial or financial relationships that could be construed as a potential conflict of interest.
