# Supplementary material for: Tumor Mutational Burden Determined by Panel Sequencing Predicts Survival After Immunotherapy in Patients With Advanced Gastric Cancer
Source: Front Oncol. 2020 Mar 13;10:314. doi: 10.3389/fonc.2020.00314 (PMC7082319; doi:10.3389/fonc.2020.00314)
Supplement: Supplementary file 1 [file Data_Sheet_1.pdf]

Table S1. Characteristics of biopsy specimens.
Table S2. List and genomic regions of target genes in Oncomine Comprehensive Assay v3 (provided in another file).
Table S3. List and genomic regions of target genes in Oncomine Tumor Mutation Load Assay (provided in another file).
Figure S1. Correlation between TMB and PD-L1 CPS.
The overall linear regression line (blue) is plotted, with the grey region showing 95% confidence interval. Spearman correlation analysis was used to investigate the relationship between two variables.
Table S4. Clinicopathologic factors associated with tumor mutational burden.
Table S5. Clinicopathologic factors according to molecular subtypes of gastric cancer.
Table S6. Univariate Cox-regression analysis for progression-free survival.
Figure S2. ROC curve and AUC of each of the indicated biomarkers (A) and their combination (B) based on PFS.
Table S7. Genes and size of panel sequencing for tumor mutational burden and their cut-off points.

Table S1. Characteristics of biopsy specimens.

**Site**	**Number of cases**
**- Stomach**	56
**- Duodenum**	1
**- Esophagus**	1
**- Ileum**	1
**- Liver**	1
**- Mesocolon**	1
**- Peritoneum**	1
**- Skin**	1
**Chemotherapy**	
**- before therapy**	46
**- after therapy**	17
**Immunotherapy**	
**- before therapy**	58
**- after therapy**	5

Figure S1. Correlation between TMB and PD-L1 CPS.

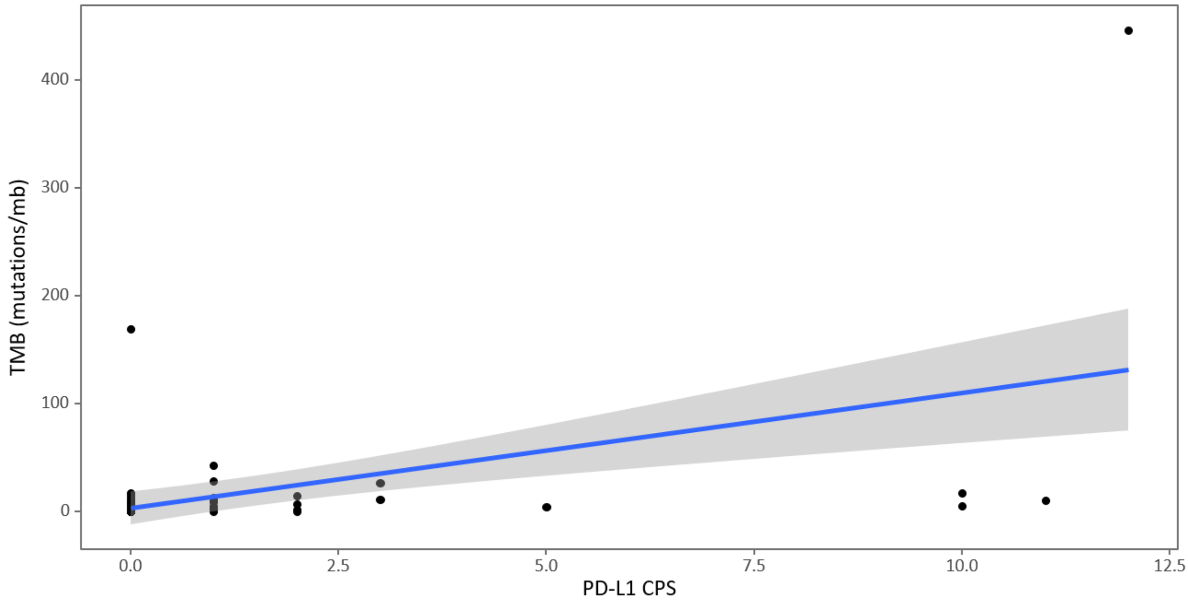

The overall linear regression line (blue) is plotted, with the grey region showing 95% confidence interval. Spearman correlation analysis was used to investigate the relationship between two variables.

Abbreviations: TMB, tumour mutational burden; PD-L1 CPS, programmed death-ligand 1 combined positive score

Table S4. Clinicopathologic factors associated with tumor mutational burden.

	Median value of TMB (range)	p-value
**Sex**		0.0947
**- F**	3.38 (0-14.31)	
**- M**	5.19 (0-445.8)	
**Age**		**0.0014**
**- <65 years**	3.38 (0-169.32)	
**- ≥65 years**	9.39 (2.52-445.8)	
**MSI status**		**<0.001**
**- MSI**	21.93 (7.58-445.8)	
**- MSS**	3.42 (0-169.32)	
**PD-L1**		0.0503
**- Negative**	3.42 (0-169.32)	
**- Positive**	5.24 (0-445.8)	
**Chemotherapy**		0.0218
**- Chemo-naïve**	3.42 (0-169.32)	
**- Chemo-refractory**	8.43 (0.84-445.8)	
**Response**		0.04
**- CR/PR**	7.58 (0-445.8)	
**- SD**	2.94 (0-13.45)	
**- PD**	4.22 (0-169.32)	

TMB, tumor mutational burden; MSI, microsatellite instability; MSS, microsatellite-stable; PD-L1, programmed death-ligand 1; CR, complete response; PR, partial response; SD, stable disease; PD, progressive disease.

p<0.05 in bold

Table S5. Clinicopathologic factors according to molecular subtypes of gastric cancer.

	EBV (n=4)	MSI (n=6)	CIN (n=23)	TP53^{+}GS^{-} (n=6)	GS (n=24)
**Age (med, range)**	67 (52-71)	74.5 (66-82)	55 (29-74)	46.5 (38-64)	53 (32-71)
**Sex (M:F)**	4:0	6:0	13:10	4:2	10:14
**Pathology**					
**- TADC**	4 (100%)	6 (100%)	20 (87%)	4 (66.6%)	17 (70.8%)
**- SRC**	0	0	2 (8.7%)	1 (16.7%)	7 (29.2%)
**- NED**	0	0	1 (4.3%)	1 (16.7%)	0
**PD-L1**					
**- Positive**	3 (75%)	4 (66.7%)	12 (52.2%)	2 (33.3%)	5 (20.8%)
**- Negative**	1 (25%)	2 (33.3%)	11 (47.8%)	4 (66.7%)	19 (79.2%)
**Median TMB (range)**	5.06 (4.23-9.32)	21.92 (7.58-445.8)	5.19 (0-26.15)	8.01 (4.23-169.32)	2.12 (0-10.93)
**- High**	0	4 (66.7%)	2 (8.7%)	2 (33.3%)	0
**- Low**	4 (100%)	2 (33.3%)	21 (91.3%)	4 (66.7%)	21 (100%)
**Response**					
**- CR/PR**	2 (50%)	5 (83.3%)	3 (13%)	2 (33.3%)	1 (4.2%)
**- SD/PD**	2 (50%)	1 (16.7%)	20 (87%)	4 (66.7%)	23 (95.8%)
**Median PFS (month)**	4.32	12.05	2.6	3.07	1.27

EBV, Epstein-Barr virus; MSI, microsatellite instability-high; CIN, chromosomal instability; GS, genomically stable; TADC, tubular adenocarcinoma; SRC, signet ring cell carcinoma; NED, adenocarcinoma with neuroendocrine differentiation; PD-L1, programmed death-ligand 1; TMB, tumor mutational burden; CR, complete response; PR, partial response; SD, stable disease; PD, progressive disease; PFS, progression-free survival.

Table S6. Univariate Cox-regression analysis for progression-free survival.

	Hazard ratio	95% CI	p-value
**Male vs. Female**	0.69	0.40-1.19	0.182
**Age (continuous)**	0.98	0.96-1.00	0.108
**EBV positive vs. negative**	1.08	0.39-3.03	0.877
**TMB-high vs. TMB-low**	0.32	0.12-0.90	**0.031**
**MSS vs. MSI**	5.17	1.24-21.53	**0.024**
**PD-L1 positive vs. negative**	0.84	0.48-1.46	0.530
**Response (SD/PD) vs. (CR/PR)**	6.34	2.61-15.44	**<0.001**
**ECOG PS ≤ 1 vs. > 1**	0.39	0.21-0.73	**0.003**
**Previous Gastrectomy Yes vs. No**	0.93	0.54-1.61	0.789
**Previous line of treatment ≤2 vs. >2**	0.88	0.51-1.53	0.647
**Peritoneal carcinomatosis Yes vs. No**	1.60	0.78-3.31	0.202

CI, confidence interval; EBV, Epstein-Barr virus; TMB, tumor mutational burden; MSS, microsatellite-stable; MSI, microsatellite instability-high; PD-L1, programmed death-ligand 1; SD, stable disease; PD, progressive disease; CR, complete response; PR, partial response; ECOG PS, Eastern Cooperative Oncology Group performance status.

$p<0.05$ in bold

Figure S2. ROC curve and AUC of each of the indicated biomarkers (A) and their combination (B) based on PFS.

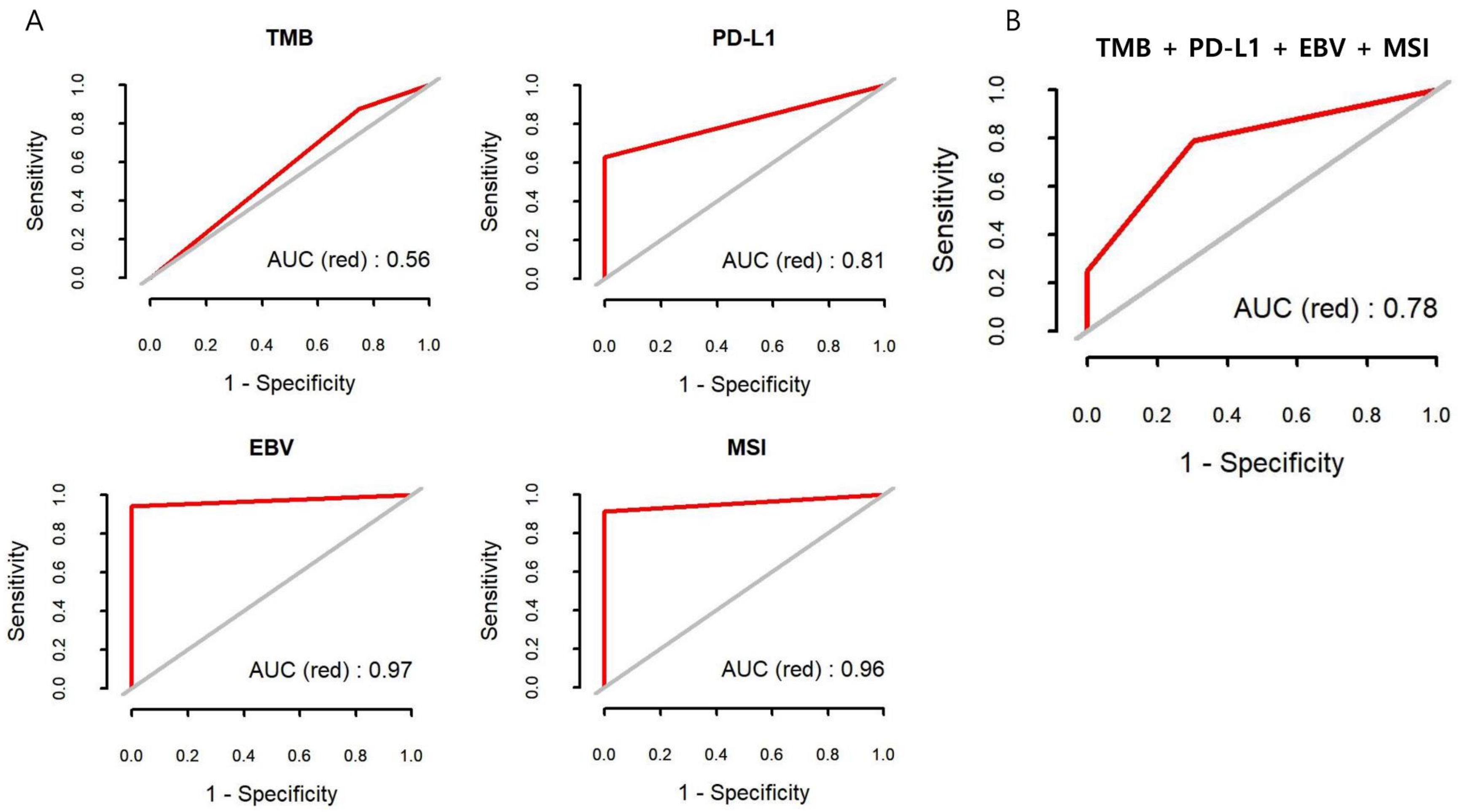

Abbreviations: ROC, receiver operating characteristic; AUC, area under the curve; TMB, tumour mutational burden; PD-L1, programmed death-ligand 1; EBV, Epstein–Barr virus; MSI, microsatellite instability

Table S7. Genes and size of panel sequencing for tumor mutational burden and their cut-off points.

Cancer type	Drug	TMB panel (number of gene, size)	Cut-off (mt/mb)	Percentile	RR	PFS (months)	OS (months)	Reference
Solid tumor	Various IO therapies	F1 (~315 genes, ~1.2mb)	20	90	58% vs. 20%	12.8 vs. 3.3	Not reached vs. 16.3	[1]
Solid tumor	NA	SSXT (592 genes, 1.4mb)	17	92.3	NA	NA	NA	[2]
Solid tumor	Various IO therapies	IMPACT v3 (468 genes, 1.22mb)	8.8	80	NA	PFS advantage	OS advantage	[3]
NSCLC	Various IO therapies	IMPACT v1~3 (~468 genes, ~1.22mb)	7.4	50	38.6% vs. 25.1%	NA	NA	[4]
NSCLC	Nivolumab + ipilimumab	F1 (324 genes, 0.8mb)	10	50	44% vs. 12%	7.1 vs. 2.6	NA	[5]
NSCLC	Nivolumab ± ipilimumab	F1 (324 genes, 0.8mb)	10	50	45.3% vs. 24.6%	7.1 vs. 3.2	NA	[6]
NSCLC	Atezolizumab	F1 (315 genes, 1.2mb)	9.9	50	25% vs. 14%	HR 0.64	HR 0.87	[7]
NSCLC	Atezolizumab	F1 (315 genes, 1.2mb)	9.9	50	20% vs. 4%	7.3 vs. 2.8	16.2 vs. 8.3	[8]
BLCA	Atezolizumab	F1 (315 genes, 1.2mb)	16	75	NA	NA	OS advantage	[9]
BLCA	Atezolizumab	F1 (NA)	9.65	50	NA	NA	HR 0.68	[10]
GC	NA	CS (404 genes, 2.3mb)	10.5	89	NA	NA	NA	[11]
GC	NA	SSXT (592 genes, 1.4mb)	17	93.1	NA	NA	NA	[12]
GC	Nivolumab	OCA v3 (161 genes, 0.39mb)	10	41	22%	1.4 vs. 2.3	NA	[13]
**GC**	**Pembrolizumab or nivolumab**	**TML (409 genes, 1.7mb)**	**14.31**	**87.3**	**50% vs. 16.4%**	**13.4 vs. 2.1 HR 0.32, p=0.023**	**16.1 vs. 4.8 HR 0.47, p=0.149**	**Present study**
**GC**	**Pembrolizumab or nivolumab**	**TML (409 genes, 1.7mb)**	**10.6**	**80**	**38.5% vs. 16%**	**2.6 vs. 2.3 HR 0.53, p=0.08**	**6.4 vs. 4.8 HR 0.63, p=0.22**	**Present study**

TMB, tumor mutational burden; RR, response rate; PFS, progression-free survival; OS, overall survival; NSCLC, non-small cell lung cancer; BLCA, bladder urothelial carcinoma; GC, gastric carcinoma; IO therapy, immune-oncologic therapy; F1, FoundationOne panel; SSXT, a custom-designed SureSelect XT assay; IMPACT, IMPACT MSKCC panel; CS, Illumina based-CancerScan; OCA, Oncomine Comprehensive Assay; TML, Oncomine Tumor Mutation Load Assay; HR, hazard ratio; NA, not available

REFERENCES

1. Goodman AM, Kato S, Bazhenova L et al. Tumor Mutational Burden as an Independent Predictor of Response to Immunotherapy in Diverse Cancers. Mol Cancer Ther 2017; 16: 2598-2608.
2. Vanderwalde A, Spetzler D, Xiao N et al. Microsatellite instability status determined by next-generation sequencing and compared with PD-L1 and tumor mutational burden in 11,348 patients. Cancer Med 2018; 7: 746-756.
3. Samstein RM, Lee CH, Shoushtari AN et al. Tumor mutational load predicts survival after immunotherapy across multiple cancer types. Nat Genet 2019; 51: 202-206.
4. Rizvi H, Sanchez-Vega F, La K et al. Molecular Determinants of Response to Anti-Programmed Cell Death (PD)-1 and Anti-Programmed Death-Ligand 1 (PD-L1) Blockade in Patients With Non-Small-Cell Lung Cancer Profiled With Targeted Next-Generation Sequencing. J Clin Oncol 2018; 36: 633-641.
5. Carbone DP, Reck M, Paz-Ares L et al. First-Line Nivolumab in Stage IV or Recurrent Non-Small-Cell Lung Cancer. N Engl J Med 2017; 376: 2415-2426.
6. Hellmann MD, Ciuleanu TE, Pluzanski A et al. Nivolumab plus Ipilimumab in Lung Cancer with a High Tumor Mutational Burden. N Engl J Med 2018; 378: 2093-2104.
7. Kowanetz M, Zou W, Shames DS et al. Tumor mutation load assessed by FoundationOne (FM1) is associated with improved efficacy of atezolizumab (atezo) in patients with advanced NSCLC. Annals of Oncology 2016; 27.
8. Kowanetz M, Zou W, Shames D et al. Tumor Mutation Burden (TMB) is Associated with Improved Efficacy of Atezolizumab in 1L and 2L+NSCLC Patients. Journal of Thoracic Oncology 2017; 12: S321-S322.
9. Rosenberg JE, Hoffman-Censits J, Powles T et al. Atezolizumab in patients with locally advanced and metastatic urothelial carcinoma who have progressed following treatment with platinum-based chemotherapy: a single-arm, multicentre, phase 2 trial. Lancet 2016; 387: 1909-1920.
10. Powles T, Duran I, van der Heijden MS et al. Atezolizumab versus chemotherapy in patients with platinum-treated locally advanced or metastatic urothelial carcinoma (IMvigor211): a multicentre, open-label, phase 3 randomised controlled trial. Lancet 2018; 391: 748-757.
11. Cho J, Ahn S, Son DS et al. Bridging genomics and phenomics of gastric carcinoma. Int J Cancer 2019.
12. Weinberg BA, Xiu J, Hwang JJ et al. Immuno-Oncology Biomarkers for Gastric and Gastroesophageal Junction Adenocarcinoma: Why PD-L1 Testing May Not Be Enough. Oncologist 2018; 23: 1171-1177.
13. Mishima S, Kawazoe A, Nakamura Y et al. Clinicopathological and molecular features of responders to nivolumab for patients with advanced gastric cancer. J Immunother Cancer 2019; 7: 24.
